# Interactions among talker sex, masker number, and masker intelligibility in speech-on-speech recognition

**DOI:** 10.1121/10.0003051

**Published:** 2021-01

**Authors:** Mathew Thomas, John J. Galvin, Qian-Jie Fu

**Affiliations:** 1 Department of Head and Neck Surgery, David Geffen School of Medicine, 10833 Le Conte Avenue, University of California, Los Angeles, Los Angeles, California 90095, USA; 2 House Ear Institute, 2100 West 3rd Street, Suite 111, Los Angeles, California 90057, USA mathewthomas@g.ucla.edu, jgalvin@hei.org, qfu@mednet.ucla.edu

## Abstract

In competing speech, recognition of target speech may be limited by the number and characteristics of maskers, which produce energetic, envelope, and/or informational masking. In this study, speech recognition thresholds (SRTs) were measured with one, two, or four maskers. The target and masker sex was the same or different, and SRTs were measured with time-forward or time-reversed maskers. SRTs were significantly affected by target-masker sex differences with time-forward maskers, but not with time-reversed maskers. The multi-masker penalty was much greater with time-reversed maskers than with time-forward maskers when there were more than two talkers.

## 1. Introduction

Segregating target from masker speech may be limited by energetic, envelope, and/or informational masking (e.g., Brungart, 2001; Brungart *et al.*, 2001; Stone and Canavan, 2016). Energetic masking describes the loss of target information due to the spectro-temporal overlap with the masker. Envelope masking describes the deleterious effects of envelope interference even when the spectral overlap between target and masker is reduced (e.g., Fogerty *et al.*, 2016; Stone and Canavan, 2016; Jennings and Chen, 2020). Informational masking in the context of this study is restricted to lexical interference (i.e., both the target and masker speech are intelligible) and talker characteristics (e.g., talker sex, the number of maskers). Envelope masking is comprised of some aspects of energetic and/or informational masking (excluding lexical interference). The effects of energetic, envelope, and informational masking may increase with the number of competing talkers (the “multi-masker penalty” described by Durlach, 2006). Normal-hearing (NH) listeners may be able to take advantage of instantaneously favorable signal-to-noise ratios (SNRs) in the spectro-temporal dips when there is only one speech masker. However, as the number of talkers increase, such “dip-listening” or target “glimpsing” becomes more difficult due to increased energetic and envelope masking. Although NH listeners may be able to hear some words in competing speech, increasing the number of talkers may negatively affect listeners' ability to distinguish target from masker words, especially when the target and masker have the same sex. This uncertainty likely increases the effect of informational masking (Brungart *et al.*, 2001; Durlach *et al.*, 2003; Kidd *et al.*, 2016).

Regardless of the number of masker talkers, recognition of the target speech is most difficult when the masker speech is intelligible, co-located with the target, and the same sex as the target (Kidd *et al.*, 2016). The effects of target-masker sex cues for segregation of competing speech have been well documented in NH listeners (e.g., Brungart, 2001; Brungart *et al.*, 2001; Rennies *et al.*, 2019). Large amounts of masking release (MR) have been reported when target and masker sex cues are different for 1-talker [10.2 dB in Cullington and Zeng (2008)] and 2-talker maskers [8.4 dB in Cullington and Zeng (2008); 9.0 dB in Brown *et al.* (2010); 14.4 dB in Rennies *et al.* (2019)]. Kidd *et al.* (2016) argued that large amounts of MR due to talker sex differences was primarily due to a reduction in informational masking. Kidd *et al.* (2016) also found that segregation of competing speech could be significantly improved by using unintelligible speech maskers (i.e., time-reversed speech). Again, the authors argued that the large MR with time-reversed speech was primarily due to a reduction in informational masking.

The combination of talker sex cues and masker speech intelligibility may further increase MR. Rennies *et al.* (2019) reported a mean MR of 14.4 dB with talker-masker sex cues and 15.3 dB with time-reversed speech. When talker-masker sex cues and time-reversed speech were combined, the mean MR increased to 19.9 dB. These effects may also interact with the number of maskers, as the amount of informational masking may increase with the number of maskers up to a certain threshold (Durlach, 2006). Yost (2017) reported that MR with spatial cues decreased as the number of symmetrically placed maskers increased from two to six. This decrease was independent of masker type, and there was almost no MR with spatial cues when there were six maskers. However, interactions among talker sex cues, masker intelligibility, and the number of maskers have not been fully explored. In the present study, speech recognition thresholds (SRTs) were measured for target speech in the presence of one, two, or four speech maskers in 10 NH adult listeners. Different combinations of target-masker sex cues were measured with different numbers of maskers. SRTs were measured for intelligible (time-forward speech) and unintelligible maskers (time-reversed speech). We hypothesized that SRTs would worsen as the number of maskers increased and that listeners would benefit from target-masker sex differences, regardless of masker intelligibility.

## 2. Methods

### 2.1 Participants

Ten NH listeners (3 males, 7 females) participated in this study (mean age at testing = 37.44 yr; range = 20–63 yr). All participants had pure tone thresholds < 20 dB hearing level (HL) at all audiometric frequencies between 250 and 8000 Hz. In compliance with the ethical standards for human participants, written informed consent was obtained from all participants before proceeding with any of the study procedures. This study was approved by the Institutional Review Board at the University of California, Los Angeles (UCLA).

### 2.2 Test materials

The matrix-style test materials were drawn from Sung Speech Corpus (Crew *et al.*, 2015, 2016), and consisted of five-word sentences constructed using randomly selected words from five categories (Name, Verb, Number, Color, and Object), each of which contained 10 monosyllable words. The target sentence was created by concatenating the randomly selected word from each category; all 50 words for the target sentence were produced by a male talker and the mean fundamental frequency (F0) across all 50 words was 106 Hz. Each masker sentence was generated by concatenating the individual words produced by one of two male talkers (mean F0s: 97 and 128 Hz) or by one of two female talkers (mean F0s: 157 and 190 Hz). For the time-forward maskers, the original 50 words were used to generate masker sentences. For time-reversed maskers, each of the words was time-reversed before generating masker sentences. All target and masker words were normalized to have the same long-term root-mean-square (RMS) amplitude. Target sentences were generated by always selecting the Name “John” (the target sentence cue word), and then randomly selecting from the 10 words in each of the remaining categories. Masker sentences were generated by randomly selecting words from each of the categories, excluding the words used for the target sentence. When there were two or more masker sentences, words were randomly selected to be different from the target sentence as well as the other masker sentences. Thus, during each test trial, target and masker sentences were comprised of different words. The duration of the words used to generate the target and masker sentences varied slightly across categories and talkers. As such, after generating the target and masker sentences, the masker sentence duration was normalized in real-time to have the same duration as the target without affecting pitch using SoundTouch software (https://gitlab.com/soundtouch/soundtouch).

### 2.3 Test conditions

SRTs, defined as the SNR that produced 50% correct word recognition, were adaptively measured. Instead of using all five words as the keywords (Rennies *et al.*, 2019), two target keywords (randomly selected from the Number and Color categories) were embedded in a five-word carrier sentence uttered by the male target talker. Since both the target and masker sentences had the same syntactic structure, different cues, such as a specific voice (e.g., Rennies *et al.*, 2019) or a specific cue word (e.g., Brungart *et al.*, 2001) are needed to cue the target sentence. In the present study, the Name “John” was used to cue listeners to the target sentence. Only two keywords were targeted, similar to many previous studies that used the coordinate response matrix (CRM) test to study masking effects (e.g., Brungart *et al.*, 2001). A greater number of keywords might differently affect working memory among listeners (Liu *et al.*, 2019), introducing an extra variable that might affect segregation of competing speech. Finally, note that while the syntax was the same for all target and masker sentences with the matrix sentences, the semantic properties may have differed across sentences, as some may have been more plausible than others. However, Calandruccio *et al.* (2018) found that the semantic “meaningfulness” of maskers did not increase masking when the syntax was fixed, as in the present study.

Recognition of the target keywords was measured in the presence of one or more masker sentences. The masker quantity was one, two, or four voices, with different combinations of male and female talkers. In this study, we abbreviate the target-masker sex cue conditions using the abbreviations from Brungart *et al.* (2001): T represents the target (male talker), S indicates that the masker talker sex (male) was the same as the target, and D indicates that the masker talker sex (female) was different from the target. Six target-masker sex cue conditions were tested: one female masker (TD), one male masker (TS), two female maskers (TDD), two male maskers (TSS), one male and one female masker (TSD), and two male and two female maskers (TSSDD). For each of the target-masker sex cue conditions, SRTs were measured with time-forward and time-reversed masker sentences.

**Fig. 1.**
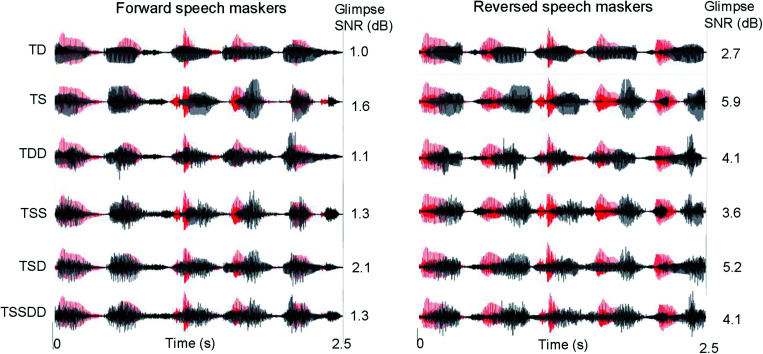
Illustration of the target-masker conditions with time-forward and time-reversed maskers. The SNR for each condition is 0 dB. The target sentence (red) is the same in all panels. The masker sentences (black) are different across the target-masker conditions; however, within each condition, the masker sentences are the same for the time-forward and time-reversed speech. The glimpsing SNR (calculated similarly to Rennies *et al.*, 2019) is shown to the right of each waveform.

Figure 1 illustrates the different masker conditions: The same target sentence is shown in red, and different masker sentences are shown for the different masker conditions in black (note that the same sentence is shown for the time-forward and time-reversed conditions within each masker condition). The density of the masker appears to increase with the number of masker talkers. With the time-reversed masker, the onset of the target words was generally less masked, potentially allowing for better glimpsing of the target sentence than with the time-forward masker (Rennies *et al.*, 2019), as illustrated in the right side of Fig. 1. Differences in energetic masking between the time-forward and time-reversed maskers were calculated similarly to Rennies *et al.* (2019). The SNR between the target and maskers across the entire sentence was 0 dB. The RMS amplitude for the target and combined maskers was calculated separately by integrating over a period of 300 ms with 20-ms sliding windows, which captured the loudness change over time, similar to a volume unit (VU) meter with its standard 300-ms integration time. After amplitude extraction, a “glimpse” of the target was defined to be available when the target RMS amplitude exceeded the combined masker RMS amplitude (i.e., when the target is louder than the combined maskers according to the VU meter). Note that this approach was somewhat different from Rennies *et al.* (2019), who extracted amplitude from 128 frequency bands with a 20-ms time window. The mean glimpsing SNR (calculated as the averaged SNRs during glimpsing moments across the entire sentence) is shown at the right of each target-masker combination. Values were much higher with time-reversed speech, suggesting less energetic masking. Note that the glimpsing SNR was only calculated for the stimuli shown in Fig. 1. However, we expect that the pattern of results would be quite similar for other combinations of target and masker sentences given that stimuli were all monosyllable words of similar duration.

**Mm. 1.** Audio file. This is a file of type “mp3” (715 KB). First the target sentence produced by the male target talker is played without any masker; this is the target sentence for all subsequent examples. Next, the TD, TS, TDD, TSS, TSD, and TSSDD conditions are played; in each condition, the target is paired with the time-forward masker first and then with the time-reversed masker.

### 2.4 Testing procedures

Stimuli were generated and diotically delivered to headphones (Sennheiser HDA 200) via audio interface (Edirol UA-25) connected to a mixer (Mackie 402). Due to the expected wide range in SRTs, a fixed overall presentation level (65 dB) was used instead of a fixed target level to avoid loud presentation levels with highly negative SNRs. Once the target and masker sentences were combined according to a specific SNR, the overall presentation level was further adjusted to have the same long-term RMS level.

During each trial, target and masker sentences were presented at the designated SNR; the initial SNR was 10 dB. Note that the SNR was calculated between the target sentence and the combined masker sentences. Participants were instructed to listen to the target sentence (produced by the male target talker and cued by the name “John”) and then click on one of the 10 response choices for each of the Number and Color categories; no other selections could be made for the remaining categories, which were greyed out. If the participant correctly identified both keywords, the SNR was reduced by 4 dB (initial step size); if the participant did not correctly identify both keywords, the SNR was increased by 4 dB. After two reversals, the step size was reduced to 2 dB. The SRT for each test run was calculated by averaging the last six reversals in SNR. If there were fewer than six reversals within 20 trials, the test run was discarded and another run was executed. Two to three test runs were completed for each condition and the SRT was averaged across runs. The target-masker sex cue, masker number (TD, TS, TDD, TSD, TSS, TSSDD), and masker intelligibility (time-forward, time-reversed) conditions were randomized within and across participants.

## 3. Results

**Fig. 2.**
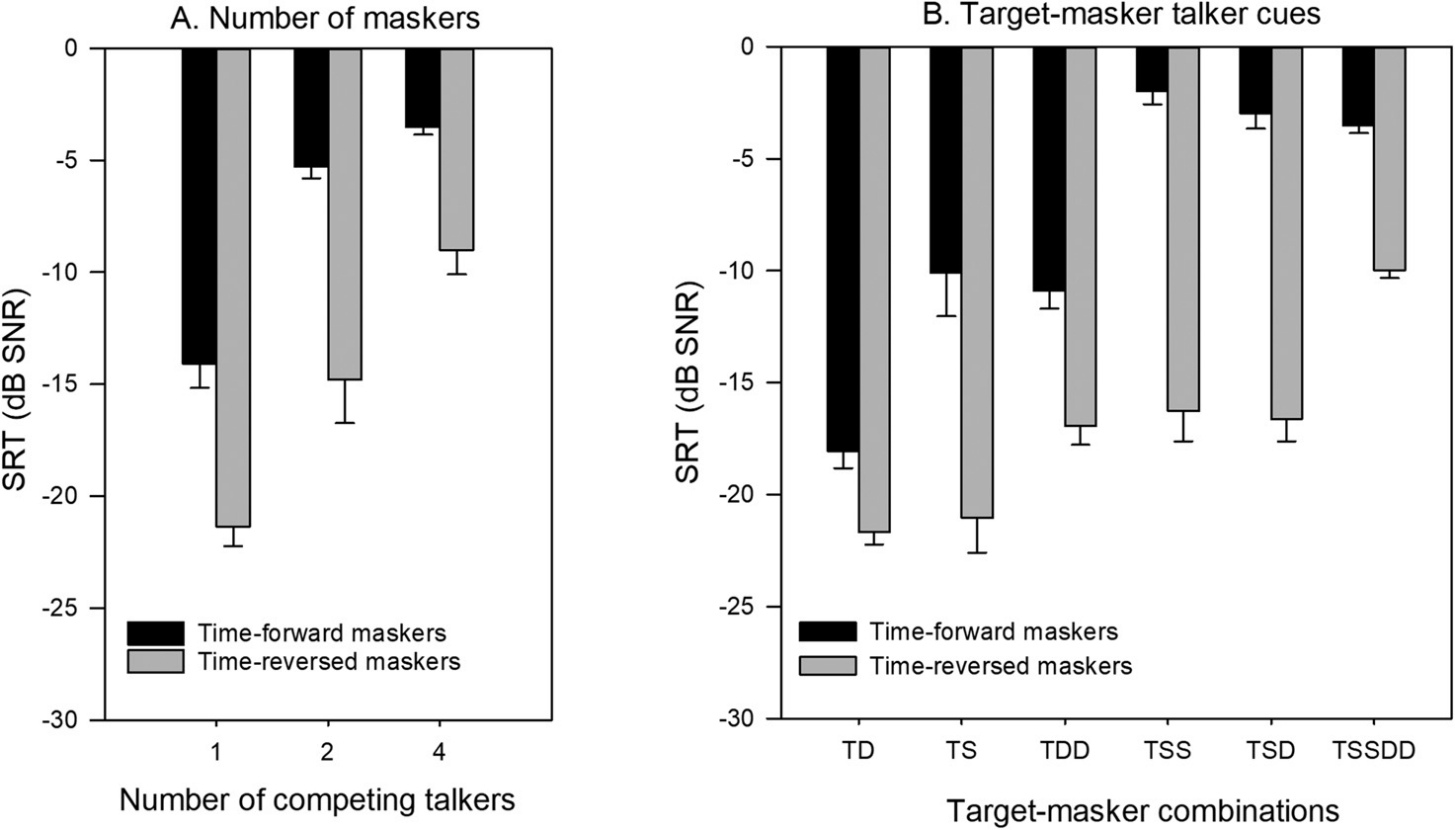
(A) SRTs as a function of the number of competing maskers for the time-forward and time-reversed maskers. (B) SRTs as a function of the individual target-masker conditions for the time-forward and time-reversed maskers. In all panels, the error bars show the standard error.

Figure 2(A) shows mean SRTs as a function of number of competing talkers for the time-forward and time-reversed maskers. Note that the 1-talker masker data represent the mean scores averaged across the TS and TD conditions; the 2-talker masker data represent the mean scores averaged across the TDD, TSD, and TSS conditions; and the 4-talker masker data represent the mean score for the TSSDD condition. For time-forward maskers, mean SRTs gradually worsened from –14.1 dB to –3.5 dB as the number of competing talkers increased from 1 to 4. For time-reversed maskers, mean SRTs gradually worsened from –21.4 dB to –9.0 dB as the number of masker talkers increased from 1 to 4. A repeated measure analysis of variance (RM ANOVA) was performed on the SRT data, with number of competing talkers (1, 2, 4) and masker intelligibility (time-forward, time-reversed) as factors. Results showed significant effects for number of competing talkers [F(2, 18) = 192.6, p < 0.001] and masker intelligibility [F(1, 18) = 303.8, p < 0.001]; there was a significant interaction [F(2, 18) = 13.5, p < 0.001].

Figure 2(B) shows mean SRTs as a function of the target-masker cue conditions for time-forward and time-reversed maskers. For time-forward maskers, the best (lowest) mean SRT was observed for TD (–18.1 dB) and the worst (highest) mean SRT was observed for TSS (–2.0 dB). For time-reversed maskers, the best mean SRT was observed for TD (–21.7 dB) condition and the worst mean SRT was observed for TSSDD (–10.0 dB). A RM ANOVA was performed on the SRT data, with target-masker cue conditions (TD, TS, TDD, TSD, TSS, TSSDD) and masker intelligibility (time-forward, time-reversed) as factors. Results showed significant effects for target-masker cue conditions [F(5, 45) = 61.4, p < 0.001] and masker intelligibility [F(1, 45) = 415.3, p < 0.001]; there was a significant interaction [F(5, 45) = 13.9, p < 0.001]. *Post hoc* Bonferroni pairwise comparisons showed that SRTs were significantly better with the time-reversed than with time-forward maskers for all target-masker cue conditions. For the time-forward maskers, SRTs were significantly better with the TD masker than with the remaining masker conditions (p < 0.05 in all cases) and significantly better with the TS and TDD maskers than with the TSS, TSD, and TSSDD maskers (p < 0.05 in all cases). For the time-reversed maskers, SRTs were significantly poorer with the TSSDD masker than with the remaining maskers (p < 0.05 in all cases); SRTs were significantly better with the TD and TS maskers than with TDD, TSS, and TSD maskers (p < 0.05 in all cases).

**Fig. 3.**
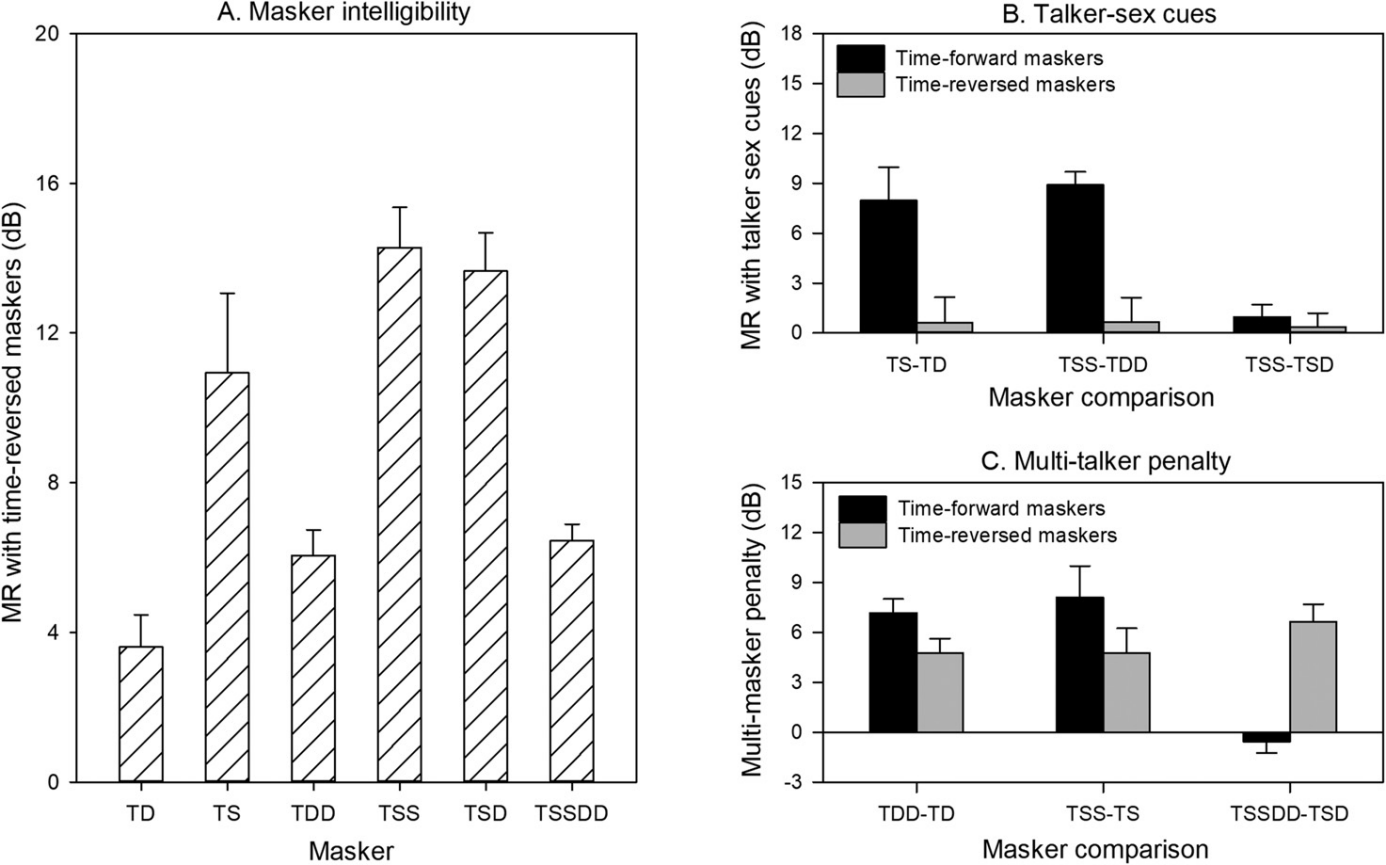
(A) Masking release (MR) due to masker intelligibility (time-forward–time-reversed SRTs) for the different target-masker combinations. (B) MR due to target-masker sex differences. (C) Multi-masker penalty for time-forward and time-reversed maskers as the number of maskers was doubled. In all panels, the error bars show the standard error.

Figure 3(A) shows MR due to masker intelligibility (the difference in SRTs between the time-forward and time-reversed maskers). A Freidman RM ANOVA on ranked data showed a significant effect of target-masker condition (χ^2^ = 25.1; p < 0.001). *Post hoc* Tukey pairwise comparisons showed that MR was significantly larger for TSD than for TD, TDD, or TSSDD (p < 0.05 in all cases), with no significant difference among the remaining target-masker conditions. Figure 3(B) shows MR according to target-masker sex cues. A RM ANOVA, with target-masker sex cue (TS-TD, TSS-TDD, TSS-TSD) and masker intelligibility (time forward, time reversed) as factors, showed significant effects for talker-masker sex cue [F(2, 18) = 6.2, p < 0.001] and masker intelligibility [F(1, 18) = 39.1, p < 0.001]; there was a significant interaction [F(2, 18) = 5.1, p = 0.017]. *Post hoc* Bonferroni pairwise comparisons showed that MR was significantly larger for the time-forward than time-reversed maskers for the TS-TD and TSS-TDD comparisons (p < 0.05 in both cases), but not for the TSS-TSD comparison.

Figure 3(C) shows the multi-masker penalty as the number of maskers was doubled. A RM ANOVA, with masker number comparison (TD vs TDD, TS vs TSS, TSD vs TSSDD) and masker intelligibility (time forward, time reversed) as factors, showed a significant effect for masker number comparison [F(2, 18) = 5.7, p = 0.012], but not masker intelligibility [F(1, 18) = 0.7, p = 0.420]; there was a significant interaction [F(2, 18) = 7.5, p = 0.004]. *Post hoc* Bonferroni pairwise comparisons showed that the multi-masker penalty with the time-forward maskers was significantly larger for the TD vs TDD and the TS vs TSS comparisons than for the TSD vs TSSDD comparison (p < 0.05 in both cases); there was no significant effect of the number of maskers for the time-reversed maskers. There was a significant difference between the time-forward and time-reversed maskers only for the TSD vs TSSDD comparison (p < 0.05).

## 4. Discussion

The present data are consistent with the hypothesis that SRTs would worsen as the number of talkers increased, regardless of masker intelligibility [Fig. 2(A)]. SRTs of the target-masker sex cue conditions for the time-forward masker data [Fig. 2(B)] were generally consistent with previous NH studies (e.g., Cullington and Zeng, 2008; Chen *et al.*, 2020). However, the present study also revealed some interesting observations in terms of interactions among masker intelligibility, talker sex cues, and the number of maskers.

Masker intelligibility contributed to both informational and energetic masking in the present study. The relatively large MR with the time-reversed maskers suggests that masker intelligibility contributed to the masking with the time-forward maskers. MR due to masker intelligibility [Fig. 3(A)] was greater when the target and masker sex was the same (TS vs TD and TSS vs TDD). Interestingly, the relatively large MR persisted even when a male and female masker was combined with the male target (TSD). When there were 2 male and female maskers (TSSDD), MR due to the masker intelligibility was reduced and was comparable to that with 1 (TD) or 2 female maskers (TDD). While the time-reversed maskers may have been unintelligible, they may have also produced less energetic masking than the time-forward maskers (Rennies *et al.*, 2019). As shown in the right column of Fig. 1, the envelope of the time-reversed maskers may have provided better glimpsing of the onset of each word in the target sentence (including the target cue word “John”). In Rennies *et al.* (2019), there was no target cue word; rather listeners were trained to attend to the target talker by listening to a 94-s sample before testing. In both cases, the reduced energetic masking with the time-reversed maskers at the onset of the first word of the sentence might allow for better perception of the target talker characteristics. Taken together, the unintelligibility of time-reversed speech may have reduced informational masking and the envelope properties may have reduced energetic masking throughout the entire duration of the target sentence.

The effect of talker sex cues on segregation of competing speech depended on whether the maskers were time forward or time reversed. Talker sex cues significantly affected SRTs with time-forward speech, but not with time-reversed speech [Fig. 2(B)]. As shown in Fig. 3(A), the effect of talker sex cues on MR due to intelligibility was quite similar for TD vs TS (difference = 7.3 dB) and TDD vs TSS conditions (difference = 8.3 dB). This suggests that talker sex cues did not interact with MR due to intelligibility. This further suggests that when masker speech is unintelligible, talker sex cues are of little use. The MR with talker sex cues with the time-forward maskers (∼8 dB) was comparable to that in Cullington and Zeng (2008), despite differences in test materials and methodology. Kidd *et al.* (2016) suggested that talker sex differences helped to reduce informational masking; consistent with this assertion, MR was greatly increased when the target and masker sex was different [Fig. 3(A)]. However, when the maskers were unintelligible (i.e., no lexical interference), listeners did not appear to benefit from talker sex cues. This suggests that talker sex cues were useful only when the maskers were intelligible, making masker intelligibility a first-order (most dominant) effect. Finally, there was no significant difference in SRTs between the TSS and TSD masker conditions, whether with the time-forward or time-reversed maskers. This is consistent with data from Calandruccio *et al.* (2017), who suggested interference by a two-talker masker is largely driven by the masker that is most similar to the target in terms of talker sex cues. However, the present data are not consistent with a related Mandarin Chinese study that showed a significant difference in SRTs (4.6 dB) between the TSD and TSS conditions (Chen *et al.*, 2020). Differences between these studies may be explained by recent findings that show that talker sex cues may allow for better segregation of competing speech for tonal languages such as Mandarin Chinese (Zhang *et al.*, 2020).

The multi-masker penalty further demonstrates interactions among masker intelligibility, the number of maskers, and talker sex cues [Fig. 3(C)]. When doubling the number of maskers from 1 to 2, the mean multi-masker penalty (averaged between TDD-TD and TSS-TS) was 7.7 and 4.7 dB for the time-forward and time-reversed maskers, respectively. When doubling the number of maskers from 2 to 4, there was no significant multi-masker penalty for the time-forward maskers; with the time-reversed maskers, the mean multi-masker penalty was 6.6 dB. Note that the multi-masker penalty was calculated according to the SNR difference. When the target-to-masker ratio (TMR) is considered (where the TMR is calculated relative to each masker, rather than the summed maskers as with SNR), the multi-masker penalty would increase by 3 dB when increasing the number of masker talkers from 1 to 2 or 2 to 4 [Fig. 3(C)]. However, the multi-masker penalty difference between time-forward and time-reversed maskers would be the same regardless of the calculation method (TMR or SNR). The pattern of results for time-forward maskers is similar to Cullington and Zeng (2008), who found no significant differences in SRTs between 2 and 4 maskers (similarly, TSSDD vs TSD). Mean SRTs were significantly poorer with 2 maskers than with 1 masker for both time-forward and time-reversed maskers. This pattern of results may be explained by dip-listening, in which the present NH listeners likely took advantage of favorable SNRs in the spectro-temporal gaps with only 1 competing talker. The worst SRT (–2.0 dB) was observed when there were two intelligible (time-forward) maskers with no target-masker sex differences (i.e., TSS). There was no significant difference in SRTs between the TSS and TSD conditions, whether or not the maskers were intelligible, consistent with Cullington and Zeng (2008). Taken together, the present data suggest that talker sex differences are useful only when maskers are intelligible and that the multi-masker penalty differs according to masker intelligibility.

While the findings may be somewhat idiosyncratic to the present stimuli and methods, the SRT and MR data are comparable to those from previous studies that used different stimuli [e.g., HINT sentences in Cullington and Zeng (2008); American Matrix Test sentences from Rennies *et al.* (2019)] and methods [e.g., word-in-sentence recognition in Cullington and Zeng (2008) and Rennies *et al.* (2019)]. This suggests that the effects of talker sex cues, number of talkers, and masker intelligibility are quite robust. The present study adds to the previous literature by finding that MR due to masker intelligibility diminished when there were more than two talkers (at least for the present tested conditions) and that the multi-masker penalty with more than two talkers was much greater with time-reversed maskers than with time-forward maskers. Given that the MR provided by time-reversed speech is due to both reduced energetic and informational masking, time-reversed speech may not be ideal when trying to probe the effects of masker intelligibility on the segregation of competing speech because of difficulties in distinguishing the contributions of the energetic and informational masking components.
